# *Helicobacter pylori* and epithelial mesenchymal transition in human gastric cancers: An update of the literature

**DOI:** 10.1016/j.heliyon.2023.e18945

**Published:** 2023-08-06

**Authors:** Tala M. Jamal Eddin, Shahd M.O. Nasr, Ishita Gupta, Hatem Zayed, Ala-Eddin Al Moustafa

**Affiliations:** a College of Health Sciences, QU Health, Qatar University, PO Box 2713, Doha, Qatar; b College of Medicine, QU Health, Qatar University, PO Box 2713, Doha, Qatar; c Biomedical Research Center, Qatar University, PO Box 2713, Doha, Qatar; d Oncology Department, Faculty of Medicine, McGill University, Montreal, QC, H3G 2M1, Canada

**Keywords:** Gastric cancer, *H.pylori*, Epithelial–mesenchymal transition, Cancer progression

## Abstract

Gastric cancer, a multifactorial disease, is considered one of the most common malignancies worldwide. In addition to genetic and environmental risk factors, infectious agents, such as Epstein-Barr virus (EBV) and *Helicobacter pylori* (*H.pylori*) contribute to the onset and development of gastric cancer. *H. pylori* is a type I carcinogen that colonizes the gastric epithelium of approximately 50% of the world's population, thus increasing the risk of gastric cancer development. On the other hand, epithelial mesenchymal transition (EMT) is a fundamental process crucial to embryogenic growth, wound healing, organ fibrosis and cancer progression. Several studies associate gastric pathogen infection of the epithelium with EMT initiation, provoking cancer metastasis in the gastric mucosa through various molecular signaling pathways. Additionally, EMT is implicated in the progression and development of *H. pylori*-associated gastric cancer. In this review, we recapitulate recent findings elucidating the association between *H. pylori* infection in EMT promotion leading to gastric cancer progression and metastasis.

## 1 Introduction

Gastric cancer (GC) is a multifactorial disorder and the fourth most common malignancy worldwide; it is considered the second cause of mortality in cancer patients [1,2]. Previous investigations have shown that 50% of newly recognized cases are observed in developing countries [1]. East Asia (Japan and China), Central and South America, and Eastern Europe are considered high risk countries having a ratio between 10 and 30% [1]. In contrast, low risk areas (North and East Africa, Australia, North America, New Zealand, and Southern Japan) show 15-20-fold decrease rate of occurrence compared to high-risk countries [2,3]. However, variations are not limited to geographical locations but also include age and gender [1,3]. Men are two to three times more prone to developing gastric cancer than women [1,4,5]. Conventional gastric carcinoma is detected in the population aged 45 years, where 10% of the cases are considered early onset (45 years and below) [1]. According to the WHO, GC is categorised into adenocarcinoma, undifferentiated carcinoma, and signet ring-cell carcinoma [3]. On the other hand, Lauren's classification divides GC into two subtypes: diffused, and intestinal [1,3]. GC is generally attributed to either environmental or genetic factors. Environmental elements encompass dietary intake, smoking, processed meat, and alcohol consumption, accounting for ∼50% of GC incidence. On the other hand, hereditary risk factors involve mostly alteration of the cadherin 1 gene (CDH1), which is associated with diffused gastric cancer cases; this genetic disorder is inherited in an autosomal dominant manner and represent 1–3% of GC cases [1,[4], [5], [6]]. In addition to these environmental and genetic factors, infectious viral and bacterial agents are also considered as causative factors comprising 5–16% of gastric cancer cases [6]. However, since 1994 *Helicobacter pylori* remains a class I carcinogen of GC development according to the WHO, representing 5.5% of the global cancer burden [1,6].

*Helicobacter pylori (H.pylori)* is a gram-negative pathogenic bacterium that colonizes the gastric epithelium selectively. Statistically 50% of the population frequently encounters infection with this bacterium; however, most individuals remain asymptomatic [7,8]. Epidemiologically, high frequencies of *H. pylori* are recorded in developing areas compared to developed regions [7,8]. One Japanese study has shown evidence of a positive correlation between *H. pylori* and GC [7]. Moreover, several investigations illustrated that eradication of *H. pylori* notably decreases the occurrence of premalignant lesions, which confirms the association of *H. pylori* with early gastric cancer stages [7,9]. A remarkable feature of *H. pylori* is the ability to tolerate high acidic environments since they are urease positive (able to convert urea to ammonia), making the gastric mucosa a suitable environment for their colonization [7,10]. The mode of transmission of *H. pylori* is via oral-oral or faecal-oral routes, with an increased infection rate during childhood [7]. Variability of outcomes due to this infection depends on several factors: genetic diversity that contributes to the inflammatory response, strain difference as well as the environmental impact, which contributes to the interactivity between host and pathogen [7,8]. Genetic studies revealed heterogeneity in *H. pylori's* genome, which contributes to variant virulence factors such as CagA, cagPAI, VacA, and adhesion proteins that affect the degree of infection and occurrence of GC [1,7,11]. In this regard, *H. pylori* can promote Epithelial-Mesenchymal-Transition (EMT), which is considered a hallmark of cancer invasion and metastasis in human cancers [12]. On the other hand, a recent report shows clearly that EBV can cooperate with *H. pylori* to enhance cancer progression via EMT [13].

EMT is a process in which epithelial cells undergo dramatic morphological changes. Thereby losing their polarity and converting to fibre-like structures that resemble mesenchymal cells [12,14]. In addition, this action decreases cell-cell adhesion properties and stimulates cell mobility, thus converting immobilized epithelial cells into mobilized mesenchymal ones. This acts as a key event that promotes cell invasion and metastasis [12]. Additionally, EMT driven de-differentiation increases mesenchymal features that initiate cancer invasion, stemness and metastasis in addition to chemoresistance [15]. It is interesting to note that such a process is reversable at the intermediate stage, cells can change phenotypes either to mesenchymal via EMT or to epithelial by MET (mesenchymal–epithelial transition) [16]. The process of EMT is accompanied by loss of E-cadherin and upregulation of Vimentin and N cadherin, resulting in the loss of epithelial properties and attainment of mesenchymal ones [12,17]. EMT is subclassified into three distinctive types that will be covered in our review.

Various factors such as stress, hypoxia, and pathogens like *H. Pylori* and EBV infections can promote EMT and result in GC initiation and progression [15,16]. This review aims to understand the underlying mechanisms by which *H. pylori* infection induces EMT by altering EMT-associated transcription factors, adhesion molecules, extracellular matrix components, and growth factor signaling pathways in gastric epithelial cells leading to the development of GC.

## *2* *H.Pylori's* pathogenicity and carcinogenicity

The clustering of *H. pylori* infection increases the risk of developing GC [7]. There are two major pathways implicated in *H. pylori* infection leading to intestinal-type GC, indirect and direct. While the indirect effect is attributed to the inflammatory processes associated with the infection; the direct pathway effects the molecular make-up of stomach epithelial cells, this comprises the toxic effect of virulence factors, deregulation in cell-cycle controlling genes, deficits in DNA repair systems, loss of a cell's adhesion capabilities, and epigenetic modifications [8].

Pathogenesis of *H. pylori's* infection can be grouped into four stages [18]. During stage 1, the bacteria enters and survives within the host. *H. pylori* utilizes an acid acclimation mechanism that neutralizes the acidic pH of the stomach. The process is regulated by intrabacterial urease activity, which breaks down urea into carbon dioxide and ammonia, thus promoting acid resistance by *H. pylori* [19,20]*.* Following the first stage, the bacteria moves via the flagella towards epithelial cells. For *H. pylori* to colonize the gastric mucosa, the bacteria migrates from epithelial to the basal layer driven by chemotaxis with a pH closer to 7.0. There are 4–7 polar sheathed flagella that achieve this process [21]. Once the migration occurs, in the next stage adhesin-receptor interaction takes place. Different bacterial strains express different adhesins, with the most common being, blood-antigen binding protein A (BabA) and sialic acid-binding adhesin (SabA) [22,23]. Other adhesions responsible for adaptation include neutrophil-activating protein (NAP), adherence-associated proteins (AlpA and AlpB), heat shock protein 60 (Hsp60), lacdiNAc-binding adhesin (LabA), and *H. pylori* outer membrane protein (HopZ) [24]. These adhesins bind to cellular receptors thereby strengthening binding of the bacterium within the mucosal layer and inhibiting bacterial displacement from the stomach due to forces such as peristalsis and gastric emptying. In the final stage, the bacteria secrete toxins to enhance its growth by damaging adjacent epithelial cells. The most common toxins are cytotoxin-associated gene A (CagA) and vacuolating cytotoxin A (VacA), and peptidoglycan which are considered part of *H. pylori's* virulence factors [25]. *H. pylori* employs a diverse range of mechanisms to modulate host cellular responses and signaling pathways. CagA stimulates inflammation, provokes the release of pro-inflammatory cytokines, enhances bacterial motility, and promotes the acquisition of cancer stem cell-like properties [26]. Additionally, CagA stimulates host cell growth and proliferation while inhibiting important cellular proteins. On the other hand, VacA creates pores in host cell membranes, leading to apoptosis and necrosis, hampers immune cell activity, and stimulates cytokine release; VacA also disrupts specific signaling pathways and influences cellular differentiation [26]. Together, the concerted action of these virulence factors contributes to the development of chronic inflammation, disruption of cellular processes, evasion of immune surveillance, which can potentially contribute to the progression of gastric cancer [26]. Table 1 indicates few *in-vivo* and clinical studies that were held to understand the role of these virulence factors.

**Table 1:** Studies depicting the role of *H.pylori*-related virulence factors.

Study Subject	Result	Reference
Mice	CagA utilizes glycoprotein-130 to induce signal transduction mediated by IL-6 and IL-11.	[27]
Human	A Japanese study revealed that *H.pylori*-infected patients had a significantly higher risk of developing gastric cancer.	[7]
Human	In a study conducted on stomach adenocarcinoma (STAD), it was found that patients infected with *H. pylori* exhibited elevated expression of IRF3/7.	[28]
Human	The study of human genetics has uncovered diversity within the genome of *H.pylori*, resulting in various virulence factors such as CagA, cagPAI, VacA, and adhesion proteins. These factors play a crucial role in determining the severity of infection and the likelihood of developing gastric cancer (GC).	[11]
Human	H.pylori strains exhibit variability in the presence of Cag PAI, with a notable impact on the severity of acute gastritis, gastric ulcers, and gastric cancer. The presence of the virulent factor Cag PAI (Cag+) correlates with an escalation in the severity of these conditions.	[29]

The major genes and molecular pathways implicated in *H. pylori* associated cancer initiation are.

### 2.1 CagA

The outcome of *H. pylori* infection is determined by the genetic heterogeneity present in its genome. CagA, discovered in the early 1990s, represents a crucial *H. pylori* protein, which is encoded by the Cag pathogenicity island (Cag PAI) and has a rigid correlation with peptic ulceration [7]. The clinical disease is associated with Cag PAI, a virulent contributing factor in *H. pylori*. The presence of this determinant is frequently indicated by CagA. However, not all Cag PAI strains express the terminal CagA gene product, which results in two classifications CagA-positive (CagA+) and CagA-negative (CagA-) [30]. *H. pylori* strains differ in the presence of Cag PAI, where the severity of acute gastritis, gastric ulcers, and gastric cancer increases when the virulent factor Cag PAI (cag+) is present [29]. The prevalence of CagA + *H. pylori* contagion is 90% in Asian countries and 60% in Western countries [7]. Furthermore, the Cag A+ category can be subclassified into East Asian-type and Western-type CagA depending on the repeat sequence Glu-Pro-Ile-Tyr-Ala (EPIYA) motifs at the N terminus of CagA [[31], [32], [33]]. The affinity of CagA to SHP-2 (Src homology 2 domain-containing tyrosine phosphatase-2) is considerably higher in East Asian-type CagA, which is considered CagA phosphorylation-dependent host cell signaling [7,34].

Consequently, East Asian-type CagA stimulates further cytoskeleton changes that increases the probability of developing gastric cancer [35]. CagA phosphorylation-independent host cell signaling involves the translocation of the bacterial protein CagA into the host gastric cell cytoplasm upon interaction with epithelial cells; whereby, CagA can alter host cell signaling via phosphorylation or translocation thereby playing a vital role in gastric carcinogenesis [36].

### 2.2 VacA toxin

VacA toxin induces intracellular vacuolation; it suppresses T-cell response to *H. pylori* [37]. The majority of *H. pylori* strains possess the VacA gene; however, significant variations in vacuolating activities were observed between different strains [38]. The variations seen in VacA gene structures within the signal (s) region, middle (m) region, and intermediate (i) region codes for these differences. The s and m regions are subclassified into s1, s2, and m1, m2, respectively [39]. The s region encodes the N terminus, while the m region encodes the C terminus. VacA s1/m1 chimeric strains (more prevalent in East Asians) trigger enhanced vacuolation than s1/m2 strains (high prevalence in Western populations); no vacuolation is seen in s2/m2 strains [40]. VacA binds to gastric epithelial cells via several receptors; the most common being the receptor-type protein tyrosine phosphatase RPTP [41]. This toxin affects host cells in several ways, such as gastric epithelial barrier interruption, inducing an inflammatory response, triggering vacuolation by disruption of the late endosomal compartment, decreasing the transmembrane potential of the mitochondria, and activating apoptosis [38,40].

### 2.3 Peptidoglycan

*H.pylori* peptidoglycan can be integrated into host cells via Nod1, which results in activating NF-κB dependant proinflammatory response [42]. However, translocated peptidoglycan of *H. pylori* stimulates other signaling pathways such as: PI3K-AKT signaling and IFN that contribute to GC development [7]. Moreover, adhesins and outer membrane proteins are considered important virulence factors, as mentioned in the section above.

Finally, it is important to highlight that *H. pylori*, play an important role in GC via the initiation of epithelial-mesenchymal transition, which is a hallmark of cancer progression. This biological event and its relation to *H. pylori* will be discussed in the section below.

## 3 Epithelial-mesenchymal transition

**Fig. 1:**
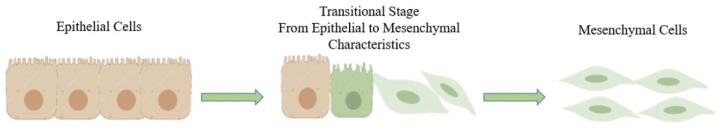
Phenotypic alterations involving loss of epithelial cell characteristics (orange to green color) and its transition to the mesenchymal cell phenotype (green color). The orange color symbolizes the characteristics of epithelial cells, which typically exhibit strong cell-cell adhesion, organized cellular morphology, and a polarized structure. These cells are associated with the maintenance of tissue integrity and specialized functions. As the color transitions from orange to green, it represents the phenotypic changes occurring during the epithelial-to-mesenchymal transition (EMT). This transition involves a loss of epithelial traits and the acquisition of mesenchymal ones, characterized by decreased cell-cell adhesion, a more elongated and spindle-shaped morphology, enhanced migratory capabilities, and increased production of extracellular matrix components.

Epithelial-mesenchymal transition (EMT) is a biological process where epithelial cells go through several biochemical changes that result in transdifferentiation into motile mesenchymal cells [43] as seen in Fig. 1.

During EMT, the basement membrane undergoes alterations and remodeling with changes in its structure and organization due to polarity loss, invasiveness, high resistance to apoptosis, migratory capacity, and elevated assembly of extra cellular matrix component (ECM) facilitating cell migration and invasion [17]. Several molecular processes play a role in establishing EMT through restructuring cytoskeletal proteins, transcription factor stimulation, secretion of ECM-degrading enzymes, specific cell-surface and distinct microRNAs expression. EMT is classified to three distinct types, which share similar genetic and biochemical origins, while having a different biological process and phenotypic programs [16]. The first type involves EMT during the embryogenesis phase where fibrosis and the invasive phenotype are not promoted. However, the embryogenesis phase includes sharing of epithelial cell plasticity characteristics which can promote the reversibility between MET and EMT. Type two EMT correlates with tissue transformation and organ fibrosis. For instance, during the repair mechanism in the wound healing stage, type 2 EM T is triggered by inflammation, however, in case of continuous respond to inflammation, organ destruction occurs [44]. While, type 3 of EMT is responsible for cancer development and metastasis and specifically appears in neoplastic cells due to genetic and epigenetic alterations that deregulate the expression of oncogenes and tumor suppressor genes [16]. It is important to note that the degree of EMT can vary, some cells conserve the epithelial characters while gaining some mesenchymal phenotypes, while others are completely transformed into mesenchymal cells. For cancer cells to possess the metastatic potential, the EMT process is controlled by epigenetic alterations of E-cadherin and β-catenin/LEF activity. This conversion provokes systemic manifestation of cancer [45]. The loss of E-cadherin is one of the significant changes that occurs during the EMT process. E-cadherin is a repressor of tumor progression by enhancing intact cell-cell contact, and preventing invasion, and metastatic diffusion [46]. In most human carcinomas, the expression of E-cadherin gene is either low or absent; the activation of E-cadherin is required to reduce tumor cell invasion and migration; studies show that loss of E-cadherin acts as a trigger for EMT and tumor metastasis [47]. The regulation of cadherins at the mRNA and protein levels occur through changes in transcriptional or translational events, protein degradation, and subcellular distribution [48]. Loss of E-cadherin in many human carcinomas is due to malfunctioning of protein production, which results from gene variation, atypical post-translational modification, or increased proteolysis [46,49].

Several in vivo and in vitro studies illustrate that neoplastic cells occupy mesenchymal phenotype and intimate mesenchymal markers such as FSP1, α-SMA, desmin, and vimentin. These markers are involved in the invasion-metastasis cascade (intravasation, moving through the circulation, extravasation, micrometastases emergence, and eventually colonization) [16,50]. Signals that induce EMT (EGF, HGF, TGF-β, and PDGF) are produced by tumor-associated stroma and are accountable for promoting functional activation in malignment cell of EMT-inducing transcription factors (Snail, Slug, zinc finger E-box bind-ing homeobox 1 (ZEB1), Twist, Goosecoid, and FOXC2) [51]. The activation of the EMT program is established through three components; intracellular signaling network (ERK, MAPK, PI3K, Akt, Smads, RhoB, β-catenin, lymphoid enhancer binding factor (LEF), Ras, and c-Fos as well as cell surface proteins such as β4 integrins, α5β1 integrin, and αVβ6 integrin) and disturbance of cell-cell adherens junctions and cell-ECM adhesions moderated by integrins [16,52].

Genetic changes, either irreversible or reversible, play a role in carcinogenesis. Epigenetic changes such as DNA and histone modifications and acetylation are examples of reversible modifications that trigger atypical gene expression in EMT during tumor progression. Hypermethylation of promoter CpG islands is the essential mechanism of tumor suppressor genes deregulation [46]. Methylation of E-cadherin promoter was observed in the majority of epithelial cancers [53]. The mechanism behind E-cadherin promoter silencing involves two models [53]. The first one suggests that Snail expression associates with E-cadherin silencing and its promoter hypermethylation. However, the second model implies that E-cadherin silencing doesn't necessitate hypermethylation of the promoter but requires further epigenetic changes, for instance histone deacetylation [46,54]. Transcriptional repressors (Snail-1, Snail2, Zeb-1, and Zeb 2) along with histone deacetylases and DNA methyltransferases account for co-repressor complexes that inhibit E-cadherin expression [46]. On the other hand, the polycomb repressive complex 1 (PRC1) protein Bmi-1 is linked with carcinogenesis and EMT [46]. Bim-1 hinders c-Myc-induced apoptosis by binding the Ink4a-Arf locus and blocking it, implying irregular cellular proliferation. Several studies demonstrated that the upregulation of Bmi-1 triggers EMT by restricting PTEN expression thus stimulating the PI3K/Akt pathway and downregulating E-cadherin expression [55,56].

### 3.1 EMT pathways and *H.pylori* in human gastric cancer

EMT is the process by which epithelial cells gain mesenchymal properties, similarly to some cells it induces cancer stem cells (CSC's) phenotype [57]. Recent studies suggest that EMT plays a substantial role in the generation of CSCs and EMT-inducing signals, which can promote the acquisition of stem-like properties in cancer cells, including self-renewal, resistance to chemotherapy and immune disruption [[58], [59], [60], [61], [62]]. CSCs are a subpopulation of cells within a tumor that can self-renew and differentiate into multiple cell types. CSCs also undergo EMT where cells acquire increased invasiveness and migratory capacity that promote in situ cancer cells to become highly invasive and disseminate to distant sites in the body, leading to metastasis [63]. The antigenic CD44 is correlated with the induction of EMT-activating transcription factors (TFs), and since gastric CSCs are CD44-positive cells, they are capable of inducing EMT [64]. Studies have shown that proteins such as: Snail-1, β-catenin, E-cadherin, vimentin, ZEB-1, and CD44 markers are EMT-interrelated in gastric cancer [65]. The presence of CD44 highly correlates with Snail-1, E-cadherin, and ZEB-1 expression. Stem cells at the level of pyloric gastric glands in the gastric epithelium can regulate Wnt pathway, which is stimulated during the EMT process; however, for the signals to be amplified, Lgr5 should be present [57,66,67].

The main mechanisms underlying EMT regulation in GC involves both transcriptional and epigenetic regulatory mechanisms. The key TFs that repress the expression of E-cadherin and trigger EMT in GC include Snail, Twist, and ZEB in GC [68]. Depending on the relation between the signals, different types of GC are developed. In intestinal GC, Snail2 and ZEB2 act synergistically. However, in diffused carcinoma, Snail1 and Snail2 act in complement [69]. Moreover, Twist expression is responsible for the degree of metastasis [70]. On the other hand, epigenetic mechanisms trigger EMT in GC via DNA methylation, histone modifications, and microRNAs. Epigenetically, in GC the promoter of CDH1 is frequently methylated, thus, CDH1 hypermethylation correlates with the degree of aggressiveness and metastasis of GC [71,72]. Histone modification (methylation or acetylation) also plays a role in GC during EMT. The transcriptional repressor enhancer of zeste homolog 2 (EZH2) is crucial for maintaining the homeostatic balance in gene expression and repression, imbalance between the two causes oncogenesis development. In GC cells, EZH2 downregulates E-cadherin due to histone H3 methylation [73,74]. On the other hand, the acetylation of H3 and H4 results in an enhanced transcription rate due to relaxed chromatin structure [75]. Moreover, microRNAs (miRNAs) act as an oncogene, or tumor suppresser and functions as a post-transcriptional regulator of genes responsible of cell differentiation, cell proliferation, and tumor growth [[76], [77], [78]]. In GC various miRNAs are deregulated such as miR-200, miR-101, miR-107, miR-221 and miR-22 [79]. MiR-200 interacts with β-catenin to suppress Wnt/β-catenin signaling, thus inhibiting tumor invasion, migration, and proliferation [80]. In GC cells, overexpression of miR-27 stimulates the Wnt pathway and results in GC cell metastasis and promotes EMT [81].

*H.pylori* colonizes directly and indirectly several targets such as fibroblasts, and epithelial cells, intraepithelial intercellular spaces, connective tissues, and extracellular matrix components and alter the mRNA expression of the genes associated with structural cell cycle regulation, growth and proliferation [82,83]. *H. pylori* interacts with fibroblasts transforming them into myofibroblasts [84], also known as cancer-associated fibroblasts (CAFs). Fibroblasts colonized by *H. pylori*, CAFs, release proinflammatory factors (COX-2, CXCL1, CXCL9, CXCL10, CXCL12, IL-6, and FSP1) and are involved in inducing tumor growth and neoplastic cell invasion [85]. Moreover, CAFs stimulate the secretion of proangiogenic factors (SDF-1, VEGF, IL-8, and FGF) and promote tumor angiogenesis, another known hallmark of cancer [86,87].

The main site for *H. pylori* growth is the gastric epithelium, the strain carrying cag-PAI induces type IV secretion system that triggers the entry of the bacterial cytokines into gastric epithelial cells; resulting in EMT progression due to phenotypic alterations in the cells [15,88]. While a positive correlation is reported between the presence of *H. pylori* and expression of TGF-β1, Snail, Slug, Twist, and vimentin mRNA [89], a negative association is observed between *H. pylori* and E-cadherin. These correlations trigger the EMT pathway (TGF-β1-induced) which play a crucial role in GC development [90]. Pathogenic *H. pylori* functions in a multidisciplinary way by upregulating soluble Heparin-binding Epidermal growth factor (HB-EGF) shedding; HB-EGF is vital for tumor progression, metastasis, and is considered a crucial factor in EMT progression especially in the gastric epithelia [91]. The overall development depends on the expression of EMT proteins “gastrin and matrix metalloproteinase-7 (MMP-7)” [92]; MMP-7 is responsible for cleaving superficial proteins, promoting cancer cell linkage, and strengthening tumor metastasis. Additionally**,** siRNA, gastrin, and MMP-7 are involved in a feedback loop to regulate EMT. In the presence of siRNA, EMT proteins are neutralized and *H. pylori*-infected cells promote EMT by upregulating the intact protein and indirectly increasing soluble HB-EGF level [92]. Additionally, CagA downregulates PDCD4 which results in an increase in the expression of TWIST1, and vimentin, while inhibiting the expression of E-cadherin [93]. This process signals a new EMT pathway in gastric cancer. By means for prevention, these two pathways can be inhibited by *H. pylori* eradication [15].

**Fig. 2:**
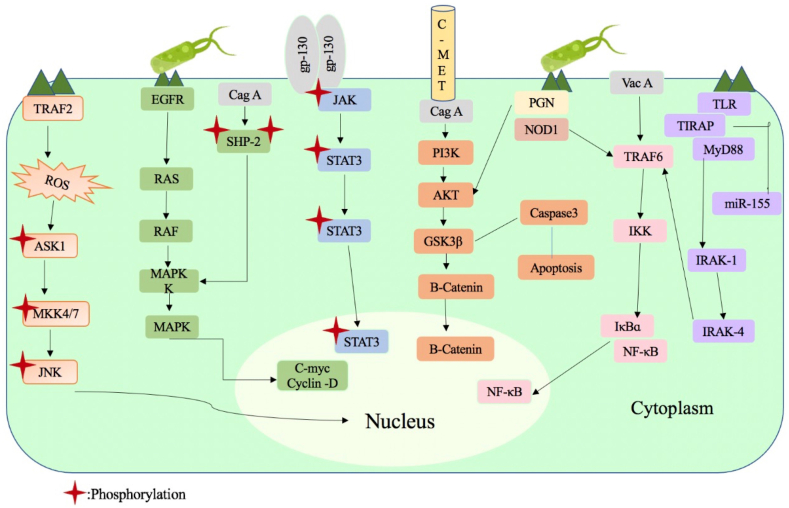
*H. pylori* induced signaling pathways to stimulate the onset and development of cancer. The figure illustrates the intricate signaling pathways induced by *Helicobacter pylori* (*H.pylori*) infection, which play a pivotal role in promoting the initiation and progression of cancer. The figure highlights several key events initiated by *H. pylori*, starting with its adherence and colonization to gastric epithelial cells. This leads to the activation of inflammatory signaling pathways, including NF-κB, MAPK, and JAK/STAT, resulting in the production of pro-inflammatory cytokines, chemokines, and reactive oxygen species. The chronic inflammation caused by *H. pylori* infection subsequently triggers DNA damage and genetic alterations in gastric epithelial cells. Concurrently, *H. pylori* stimulates cell proliferation and survival pathways, such as PI3K/Akt and Ras/Raf/MAPK, promoting uncontrolled cell growth and resistance to apoptosis.

Similar to the role of *H. pylori* in inducing EMT in GC, *H. pylori* can deregulate several molecular pathways which are vital for the onset and development of cancer and will be described in the section below (Fig. 2) [94].

#### 3.1.1 Janus kinase/Signal transducer and activator of transcription pathway

Signal transducer and activator of transcription 3 (STAT3) is responsible for angiogenesis proliferation, apoptosis, and basal homeostasis; hallmarks for cancer development including GC. STAT3 can be activated either by proinflammatory cytokines and growth factors secreted by *H. pylori* or receptor tyrosine kinases phosphorylation (JAK1, JAK2, and Src) [27,88]. From the ∼30 genes that *H. pylori* possesses, the CagA gene activates the STAT3 pathway and induces GC (Fig. 2) [95]. Studies done in mice models suggest that utilization of the glycoprotein-130 by CagA induces signal transduction by IL-6 and IL-11 [27]. IL-6 stimulates the recruitment and homodimerization of gp130, which results in a balance between the two signaling pathways (JAK/STAT and SHP2/Ras/ERK) [96]. On the other hand, IL-11 functions as an activator of gastric STAT3 in the early stages [97].

#### 3.1.2 Interferon regulatory factor signaling pathway

Type I interferon production increases during *H. pylori* infection of gastric epithelial cells, due to TLRs −7, −8, or −9 activation. Several factors such as NFκB, activator protein 1 (AP1), and IRF3/7 are responsible for type 1 interferon production. In *H. pylori* DC-SIGN receptors account for IRFs (IRF3/7) activation [98]. Moreover, the severity of *H. pylori* is determined by the presence of cytokine and chemokines stimulated by interferon type 1 produced by nucleotide-binding oligomerization domain 1 (NOD1) signaling pathway [88,99]. A study done on stomach adenocarcinoma (STAD) revealed *H. pylori* infected patients to have an increase in IRF3/7 expression [28]. IRF7-induced IFN-β production activates type I IFN, IFN- stimulated gene factor 3 (ISGF3) along with subsequent production of CXC motif chemokine ligand 10 (CXCL10) [100]. *H. pylori* infection also stimulates the IFN signaling pathway regulated by two elements (IRF 1 and STAT1), resulting in activation of NOD1 pathway which is responsible of increasing STAT1-Tyr701/Ser727 phosphorylation levels and IRF1 expression in epithelial cells. This process results in elevated chemokines production coordinated by IFN-γ induced protein 10, IFN-γ, IL-8 and NOD1 [101].

#### 3.1.3 Nuclear factor kappa B pathway

*H.pylori* activates NFκB through several factors including LPS, peptidoglycan and virulence genes (CagA). Through TLR activation, *H. pylori* regulates the level of NFκB and alters the signaling of its canonical and non-canonical pathways [102]. Both of these pathways are reactivated in B-lymphocytes; however only the canonical one is activated in epithelial cells [103]. The kinase complex IκB kinase (IKK) is activated when the epitope binds to the receptor, allowing the translocation of the canonical NFκB heterodimer of RelA/p65 and p50 thereby enhancing the breakage of inhibitor IκB phosphorylation [103,104]. In B-lymphocytes, LPS of *H. pylori* activates the pathway via NFκB inducing kinase (NIK) and IKK, where the receptor's downstream activates the IKKα and NIK (Fig. 2). Activated IKKα phosphorylates its downstream P100, responsible for p52 proteasomal breakdown [105]. Two factors (RelB and P52) act together to promote B cell survival, maturation, lymphoid organogenesis and bone metabolism. In gastric epithelial cells, CagA coordinates with intramembrane hepatocyte growth factor receptor (HGFR)/MET triggering the PI3K-Akt pathway, which turns on β-catenin and NFκB (Fig. 2). Moreover, interaction of TRAF6 and TGF-β-activating kinase 1 (TAK1) leads to CagA-induced TAK1 expression which causes upregulation of NFκB either throught the activation of IKK complex due to TAK1 phosphorylation or by CagA multimerization via the Met-PI3K-Akt signaling pathway [103].

#### 3.1.4 c-Jun proto-oncogene signaling pathway

In gastric epithelial cells, *H. pylori* infection induces apoptotic cell death through the activation of new signaling pathways “ROS/ASK1/JNK” [106]. Apoptosis signal-regulating kinase 1 (ASK1) is an enzyme produced in the presence of *H. pylori* in a ROS and cagPAI-dependent manner [107]. Intracellular ROS releases ASK1 from its binding protein Thioredoxin (TRX) and activates it [108]. Moreover, ASK1 is responsible for *H. pylori* mediated apoptosis, and JNK initiation. In a ROS-dependent manner, TAK1 regulates JNK activity positively and negatively [107]. A negative loop between ASK1 and TAK1 is responsible for the equilibrium between ASK1-induced apoptosis and TAK1-induced anti-apoptotic responses, which determine the fate of epithelial cells. When TAK1 or downstream p38 MAPK is suppressed, ASK1 is stimulated in a ROS-dependent manner resulting in downstream NFκB activation in *H. pylori* response. However, when TAK1 binds to TAB1, ASK1 is inhibited. Activation of the downstream JNK, MAPK, and p38 is regulated by the phosphorylation of MKK4, MAP2Ks, MKK3, and ASK1 [107].

#### 3.1.5 TGF-β pathway

The primary tumorigenesis transformation growth factor beta (TGF-β) is the key suppresser of epithelial cell propagation and promotes EMT through two signaling pathways. The first pathway includes Smad proteins which arbitrate TGF-β-induced EMT through ALK-5 receptor, that facilitate motility and mediates the activity of LEF and β-catenin via interaction with Smad. On the other hand, the secondary TGF-β-induced pathway involves p38 MAPK, RhoA, integrin β1–mediated signaling and the activation of latent TGF-β by αVβ6 integrin [16,[109], [110], [111], [112]].

#### 3.1.6 Phosphatidylinositol 3-kinase pathway

Activator protein 1 (AP-1) is a transcription factor that is present in Jun and Fos proteins. It is involved in the subunit of Fos (c-Fos, FosB, Fra- 1, Fra-2), Jun (c-Jun, JunD, JunB), musculoaponeurotic fibrosarcoma (MAF) and activating transcription factor (ATF) [113]. Infection with *H. pylori* are known to activate AP1 pathway through two mechanisms: cagPAI and NOD1 [114]. Type IV secretion system (T4SS) secreted by cagPAI deliver the CagA into host cells by creating pilus structure and secreting T4SS proteins. Oncogenic tyrosine kinases phosphorylate CagA to imitate host sell factor. NOD1 recognize this action and activate MAPK, NFκB, and AP1 [114].

#### 3.1.7 Mitogen-activated protein kinases (MAPK)

RAS and RAF proteins activate MARK to directly upregulate ERK. Specific ERK proteins stimulated by MEK phosphorylate the c-Myc, and Elk-1 transcription factors [115]. Upon IKK-β and cytosolic phospholipase A2 (cPLA2) phosphorylation, *H. pylori* LPS stimulates ERK, that in turn enhances the translocation of NFκB and encourages the production of COX-2 and iNOS. *H. pylori* neutrophil-activating protein (HP-NAP) stimulates cells of the immune system, since it resembles virulence factors. In human neutrophils, HP-NAP provokes ERK and p38-MAPK initiation [116]. Moreover, *H. pylori* prompts serum-responsive element (SRE) dependent gene transcription and increases c-Fos protein expression, revealing the signaling mechanism through which *H. pylori* activates ERK [116].

#### 3.1.8 Wnt/β-catenin pathway

*H.pylori* provokes gastric epithelial cell proliferation through β-catenin via three mechanisms; activation of oncogenic c-Met and epidermal growth factor receptor (EGFR), inhibition of tumor suppressor Runx3 and Trefoil factor 1 (TFF1), or by recruiting macrophages [117]. The pathway is introduced by secreted glycoproteins (Wnt1 and Wnt3a) that will attach to a receptor, Frizzled, and a co-receptor, lipoprotein receptor-related protein 5/6 (LRP5/6), promoting β-catenin detachment from its degrading complex. This complex is comprised of scaffold protein AXIN, casein kinase 1α (CK1α), tumor suppressor adenomatous polyposis coli (APC), and glycogen synthase kinase 3β (GSK3β). Thereby, β-catenin accumulates as it escapes from two processes: phosphorylation by glycogen synthase kinase 3β (GSK3β), and degradation by ubiquitin-proteasome system (UPS). The cumulation of β-catenin in the cytoplasm shifts into the nucleus, and merges with T cell factor/lymphocyte enhancer factor (TCF/LEF) [117].

Other molecular pathways that have been reported to play a role in *H. pylori* infection include HIF-1***α*** [118], BCR [119], and TLR [120] signaling pathways.

## 4 Conclusion and future perspective

This review presents a summary viewpoint on the role of *H. Pylroi* and its oncoproteins CagA, CagPAI, VacA in the initiation and progression of EMT by alteration of its main biomarkers and deregulation of several signaling pathways, mainly Interferon regulatory factor, NF-κB, PI3k, MAPK and Wnt/β-catenin. Although the role of *H. pylori* is well described in human gastric diseases especially gastric ulcer, its function in the development and/or progression of human cancer via EMT is not fully understood. Thus, we believe that developing in vitro and in vivo experimental models to unravel the underlying complex mechanisms of *H. pylori* infection in EMT can elucidate their role in cancer progression. Such advancements can potentially help identify specific therapeutic targets and pave the way for new management approaches of metastatic GC which is the major cause of cancer related death.

## Author Contributions

All authors listed have significantly contributed to the development and the writing of this article.

## Data availability statement

No data was used for the research described in the article.

## Funding statement

Open Access funding was provided by Qatar National Library.

## Institutional review board statement

Not applicable.

## Informed consent statement

Not applicable.

## Declaration of competing interest

“The funders had no role in the design of the study; in the collection, analyses, or interpretation of data; in the writing of the manuscript, or in the decision to publish the results”.
